# STARTING FROM STRENGTH: BUILDING PARTNERSHIPS IN COMMUNITY ENGAGED INTERVENTION RESEARCH FOR DEMENTIA INCLUSION

**DOI:** 10.1093/geroni/igae098.2363

**Published:** 2024-12-31

**Authors:** Sheila Novek, Heather Neale Furneaux, Eric Macnaughton, Paulina Malcolm, Andréa Monteiro, Alison Phinney

**Affiliations:** University of British Columbia, Vancouver, British Columbia, Canada; University of British Columbia, Vancouver, British Columbia, Canada; University of British Columbia, Vancouver, British Columbia, Canada; University of British Columbia, Vancouver, British Columbia, Canada; University of British Columbia, Vancouver, British Columbia, Canada; University of British Columbia, Vancouver, British Columbia, Canada

## Abstract

The majority of people with dementia live at home, often disconnected and isolated from their community. Canada’s Public Health Agency recognizes that community-based organizations are well positioned to address this issue, and through its Dementia Community Investment is advancing social innovation as a strategy for increasing inclusion, well-being, and quality of life for this growing population. The Building Capacity Project (2019-23) used asset-based community development to foster dementia inclusion through community programs and activities. Using developmental evaluation with community partners in Vancouver Canada, we identified a core set of guiding principles and promising practices for supporting dementia inclusive innovations in community programming: addressing stigma; including lived experience; organizational coaching; and social networking. Now in Phase 2 (2023-25), we are doing community-engaged intervention research, using the Evidence Based System for Innovation Support as a theoretical framework to scale out impacts with new partners in urban and rural communities across the region. As a first step, we conducted semi-structured interviews with 12 representatives from seven community organizations. Based on a thematic analysis of the interview data, we identified three areas for further capacity building for dementia inclusion: (1) reframing dementia as a shared experience; (2) growing confidence; (3) centering joy; and (4) making connections. These findings set the stage for further work as organizations identify strategies for reaching out to people with lived experience at the local level, expand opportunities for more diverse program offerings in multiple languages, and leverage opportunities for integration across municipalities, health systems, and the social services sector.

